# Editorial: Advancing biostatistics and informatics applications in mental health research

**DOI:** 10.3389/fpsyt.2026.1948173

**Published:** 2026-08-19

**Authors:** Irene Suilan Zeng, Rodger Kessler

**Affiliations:** 1 Biostatistics, Auckland University of Technology Faculty of Health and Environmental Sciences, Auckland Central, New Zealand; 2 DARTNet Institute, Aurora, CO, United States

**Keywords:** clusters and multivariate structures, national integrated data informatics system, natural language processing and clinical documentation, networks, supervised learning

## Introduction

In the history of biostatistics application in research, researchers typically start with a question or hypothesis and propose a data model. In contrast, recently emerged machine learning methods, often start with the information already available and propose hypothesis using data as the starting point. These represent the “two-cultures” paradigms of statistical modelling described by Breiman (1) as data modelling and algorithmic modelling. The conventional data modelling approach perceives data generated from an underlying probabilistic structure and focuses on estimation, interpretation, and inference. The algorithmic modelling assumes that data may not arise from an easily described specified model. It uses an algorithm to predict the response and is evaluated by measuring predictive accuracy.

When the informatics era came, we have a modern problem and the excitement of growing data in terms of its dimensionalities and volume. The growth of informatics has intensified the importance of both modelling approaches. Researchers now routinely work with high-dimensional and multimodal data, including genomics, neuroimaging, wearable sensors, electronic health records, administrative databases, and natural language text. While traditional statistical methods remain critical for causal inference and evaluation in clinical and epidemiological studies, algorithmic approaches provide powerful tools for uncovering complex relationships within increasingly large and interconnected datasets. The two-culture principle suggests that we should search for the model that provides the best solution- data modelling, algorithmic modelling, or both.

The current volume of the Research Topic “*Advancing Biostatistics and Informatics Applications in Mental health Research”* brings together 11 articles of five themes that collectively demonstrate the expanding role of advanced analytics in mental health research.

## Exploring networks, clusters and multivariate structures

Several studies demonstrate the value of multivariate approaches for characterizing complex mental health phenomena.

Chen utilized network structure learning, an algorithmic modelling method, to explore the interrelations between major depression symptoms and ambivalence over emotional expression (AEQ) and compared the symptom networks between male and female college students. They identified two clusters of comorbid symptoms in the depression-AEQ networks, discovered the bridging symptoms between two clusters and examined the gender difference in network and centrality. Notably, the study combined network methods with statistical validation procedures, illustrating productive integration of algorithmic and data modelling approaches.

Youvalari and Gökbay provided a profiling analysis of resilience and Taghi demonstrated the potential of machine learning to enrich psychological Modelling. Grounded in the five-dimensional (5D) resilience framework and used unsupervised algorithmic modelling, the authors identified hidden psychological patterns clustered into 4 archetypes (Reactive Idealists, Fragile Strivers, Hidden Reactors, Stable Withdrawers). An important innovation of the study is to combine a deep autoencoder for feature extraction, with Gaussian Mixture Model clustering; and provided narrative description of archetype profiles.

Lee et al. proposed digital phenotyping by integrating wearable Fitbit data, SOMDAY application, neuroimaging data (fNIRS), and genomic information. The periodical nature and activities of sleep was used for the multivariate pattern analysis and identification for digital phenotypes. The analytical framework conceptualized integrating the genetic vulnerability (polygenic risk scores), molecular state (gene expression), and physiological outputs (biomarkers) in the context of environmental and behavioral modulating factors captured through digital phenotyping.

The New Zealand study by Zeng et al. similarly demonstrated the value of analytical triangulation. By combining data modelling using quantile regression, path analysis, with algorithmic Bayesian network modelling within complex survey data, the authors identified consistent associations between mental wellbeing and factors such as perceived health, life satisfaction, cultural identity acceptance, and trust in educational institutions. The study also highlights methodological advances in calibrating complex survey designs through replicate-weight approaches.

## Natural language processing and clinical documentation

Advances in natural language processing (NLP) and large language models (LLMs) are increasingly influencing mental health research and clinical practice. Goga et al. conducted texts analysis using social media discussions and semantic techniques. A supporting objective of the research was to create a natural language processing (NLP) tool for classifying depression to obtain the corpus necessary for ontology creation. The innovations were automation of ontology generation from unstructured text and compared them across depressed and non-depressed, religious and non-religious groups, in concept, hierarchical and relation similarities.

Niederlohmann proposed clinical translation methods for classifying psychological session cues using a perceived-assess-dose-safeguard decision matrix and a conflict-square algorithm (PAD-S/CSA model). The study proposed a quantitative translation from less structured narrative clinical diagnosis notes to clear formulated documentation; to assist psychotherapists to provide next treatment move and tolerance threshold for the treatment. It generates a separated process notation to assist clinical reasoning and decision-making based on safety, tolerance, shame, avoidance, and functional recovery for SMI and multimorbidity.

## Using supervised learnings to test the hypotheses

Yang et al. set an example of using both algorithmic and data models to investigate the trajectory clusters of depression symptoms among hospitalized patients and test the bidirectional associations between cognitive functions and depression symptoms over time. They applied the algorithmic Gaussian Mixture Model, a weighted combination probability model of multiple Gaussian distributions, allowing flexible clustering and density estimation. Three trajectory groups were identified: Severe-Rapid Remission; Moderate-Rapid Remission; Moderate-Slow Remission.

Su et al. using data-driven algorithmic model and logistic regression to develop the final nomogram. The study has introduced SMOTETomek hybrid sampling (2), a method of generating synthetic minority-class samples and reducing class overlapping to resolve data imbalance. This study compared four machine learning models: eXtreme Gradient Boosting (XGBoost), Adaptive Boosting (AdaBoost), Random Forest (RF), and Gradient Boosting Decision Tree (GBDT), and demonstrates how prediction models can be translated into clinically interpretable tools.

## Utilizing national integrated data informatics system and public repositories

The increasing availability of integrated data systems has transformed opportunities for mental health research. INSPIRE network datahub (Bhattacharjee et al.) project using the Observational Medical Outcomes Partnership (OMOP) Common Data Model and OHDSI tools to enhance data interoperability and analytic capabilities. Grounded in the FAIR (Findable, Accessible, Interoperable, Reusable) principles, OMOP common data model is community harmonized open data standard using standardized structure to enable effective analysis. Evaluating by a data-quality index of plausibility, conformance, and completeness, the migrated longitudinal studies established a *multidimensional* landscape of mental health across African contexts.

At the national level, other studies leveraged national administrative and survey data to address population-level questions such us Czech et al. ‘s integrated pharmaceutical, healthcare, and social insurance and the New Zealand IDI-based study. These examples highlight the growing importance of secure, integrated research infrastructures for studying mental health across populations and life stages.

## Time trend analysis and projection of disease burden across multiple dimensions

Understanding temporal changes in mental health remains a major public health priority. Czech et al. triangulated several administrative data sources and provided converging evidence of increased mental health burden among youth in COVID-19. The authors used linear regression trend models, structural-break adjustment for the 2019 reporting-system change, and modified Gompertz modelling for long-term trend estimation. Their findings provided convergent evidence of substantial and sustained impacts on youth mental health. At a global scale, Zhou et al. presented decomposition analysis quantifying contributions of population growth, ageing, and epidemiological change. The projection of disease burden was through Joinpoint regression identifying trend inflection points and Sex-stratified forecasting. Both studies acknowledge and address important methodological challenges.

## Conclusion

The studies in this volume illustrate the evolving landscape of mental health research, where traditional biostatistics, machine learning, informatics, and artificial intelligence increasingly converge. Across diverse settings and data sources, researchers are applying innovative analytical approaches to investigate symptom networks, resilience profiles, digital phenotypes, clinical documentation, disease prediction, integrated data systems, and population trends.

A recurring theme is the complementary value of data modelling and algorithmic modelling. As mental health datasets continue to grow in scale and complexity, the integration of biostatistics and informatics will play an increasingly important role in advancing research, practice, and public health.
